# Perceived stress in patients with migraine: a case-control study

**DOI:** 10.1186/s10194-017-0780-8

**Published:** 2017-07-21

**Authors:** Hye-Jin Moon, Jong-Geun Seo, Sung-Pa Park

**Affiliations:** 1 Department of Neurology, Soonchunhyang University Bucheon Hospital, Bucheon, Republic of Korea; 2 Department of Neurology, School of Medicine, Kyungpook National University, 680 Gukchaebosang-ro, Jung-gu, Daegu, 41944 Republic of Korea

**Keywords:** Migraine, Perceived stress, Depression, Anxiety, Insomnia, Quality of life, Predictor

## Abstract

**Background:**

Perceived stress is the most common trigger for migraine. The objective of this study was to examine the clinical significance of perceived stress in migraine patients.

**Methods:**

This is a case-control study. Consecutive migraine patients who visited a tertiary care hospital were enrolled for this study. They completed self-reported questionnaires including Perceived Stress Scale (PSS), 12-item Allodynia Symptom Checklist (ASC-12), Migraine Disability Assessment Scale (MIDAS), Patient Health Questionnaire-9 (PHQ-9), Generalized Anxiety Disorder-7 (GAD-7), Epworth Sleepiness Scale (ESS), Insomnia Severity Index (ISI), and Migraine-Specific Quality of Life Questionnaire (MSQ). Degree of perceived stress in migraine patients was measured and compared to that in healthy controls. Predictors for perceived stress and their impact on quality of life (QOL) of migraine patients were also determined.

**Results:**

A total of 227 migraine patients were eligible for this study, including 103 (45.4%) who had chronic migraine (CM). Mean PSS score was significantly (*p* < 0.05) higher in CM patients than that in controls after adjusting for education, depression, and anxiety. Although several factors were associated with PSS score, major predictors for PSS were GAD-7 score (*β* = 0.358, *p* < 0.001), PHQ-9 score (*β* = 0.304, *p* < 0.001), ISI score (*β* = 0.154, *p* = 0.005), and CM (*β* = −0.104, *p* = 0.027). There was an inverse relationship between PSS scores and three-dimensional scores of MSQ (*p* < 0.001).

**Conclusions:**

Chronic migraine is a critical factor for perceived stress. Perceived stress affects QOL of migraine patients.

## Background

Migraine, a common disabling disease, accounts for a large proportion of non-fatal disease related burden worldwide [1]. In a review study on global burden of disease in 2013, migraine and mild-to-moderate mental disorders such as depressive and anxiety disorders were main causes of burden in this category for the Korean public [2]. Migraine has several comorbidities and modifiable risk factors. In published literature, vascular accidents, depression, anxiety, epilepsy, and sleep problems are commonly associated with migraine. Attack frequency, caffeine, medication overuse, obesity, snoring or sleep apnea, psychiatric comorbidity, and stressful life events have been suggested as modifiable risk factors for migraine complications such as vascular events and chronic migraine (CM) [3]. All these factors will reduce the quality of life (QOL) of migraine patients [3, 4]. In addition, psychiatric comorbidity and psychological distress may negatively affect the outcome of migraine patients [5].

It has been found that migraine patients have higher levels of perceived stress than healthy controls [6, 7]. In addition, identified stress levels are higher in migrainous women than those in migrainous men [6]. Moreover, stress can trigger migraine attacks. About 80% of migraine patients with identifiable triggers have reported that stress is a common trigger [8]. In a Korean hospital-based study, stress is the most common trigger for episodic migraine, followed by sleep deprivation and fatigue [9]. In addition, 57.7% of patients have indicated fatigue as a headache trigger [9]. It has been suggested that stress might be a predisposing factor in new-onset migraine [10]. Stress might also play a role in migraine chronification [11].

Although a stressful condition is likely to be associated with migraine, the level of perceived stress between episodic migraine (EM) and CM has not been delineated yet. Therefore, the first aim of this study was to determine the level of perceived stress in EM and CM patients. Factors associated with perceived stress in migraine patients have not been reported yet. If predictors for perceived stress of migraine patients can be identified, a guideline can be developed for clinicians to manage stress adequately. Therefore, the second aim of this study was to identify predictors for perceived stress in migraine patients. In addition, although it is known that migraine and comorbid disorders will reduce QOL of patients, the impact of stress on QOL has not been reported. Therefore, the third aim of this study was to delineate the impact of stress on QOL to provide information for clinicians.

## Methods

### Subjects

New patients with migraine who consecutively visited an outpatient clinic in the Department of Neurology at Kyungpook National University Hospital since April 2015 were recruited. Patients with age between 18 and 70 years were included. Patients were diagnosed by the International Classification of Headache Disorders 3rd edition, beta version [12]. Patients who had illiteracy, mental retardation, serious medical, neurological, or psychiatric disorders, and history of alcohol or drug abuse that prevented them from cooperating with us during the study were excluded. Patients who refused to fill out questionnaires and those whose diagnosis was a probable migraine were also excluded. Age- and gender- matched healthy controls were also enrolled. They were university students, office workers, teachers, or hospital employees.

### Study design

A case-control study was performed as part of a hospital-based study to examine the impact of psychiatric and psychosocial problems on migraine. This study was approved by Institutional Review Board of Kyungpook National University Hospital. Written informed consent was obtained from each subject. Subjects were interviewed. Demographic and socioeconomic data were collected. Socioeconomic data included employment status and household income (monthly income greater or less than three million KRW per month equivalent to 2800 USD). Patients were asked for their concurrent medical diseases, type of migraine, migraine chronicity, age at onset, duration of migraine, headache intensity, medication overuse headache, family history of migraine, accompanying symptoms including nausea and/or vomiting, photophobia, phonophobia, osmophobia, and allodynia. Headache intensity was measured using Visual Analog Scale (VAS). Photophobia, phonophobia, and osmophobia during migraine attacks were defined as hypersensitivity to light, sound, and certain odors, respectively. Allodynia was measured because it was associated with depression and chronic migraine in our previous study [13]. It was measured by the 12-item Allodynia Symptom Checklist (ASC-12) with a cut-off score of >2 to define allodynic patients [14].

Eligible subjects underwent several self-reported questionnaires including the Korean version of Perceived Stress Scale (PSS) [15], Migraine Disability Assessment Scale (MIDAS) [16], Patient Health Questionnaire-9 (PHQ-9) [17], Generalized Anxiety Disorder-7 (GAD-7) [18], Epworth Sleepiness Scale (ESS) [19], Insomnia Severity Index (ISI) [20], and Migraine-Specific Quality of Life Questionnaire version 2.1 (MSQ) [21]. The degree of PSS score in migraine patients was examined and compared to that in controls. Predictors for PSS score were identified and the relationship between PSS score and MSQ was determined.

### Questionnaires

#### Perceived Stress Scale (PSS)

The Korean version of the PSS-10 was used in this study [15]. It measures stress appraisal during the preceding month. It has two subscales: a negative subscale (items 1, 2, 3, 6, 9, and 10) and a positive subscale (items 4, 5, 7, and 8). These items were rated in a five-point Likert scale, ranging from 0 to 4. A higher summed score means higher perceived stress. Cronbach’s α coefficient of this scale was 0.819.

#### Migraine Disability Assessment Scale (MIDAS)

The Korean version of MIDAS includes a 5-item questionnaire. It was designed to evaluate disability during the past three months. [16] Subjects were asked to report decreased performances in domains of work/school, household work, and family/social activities. Scores were used to measure the overall level of disability: Grade I, scores of 0–5; Grade II, scores of 6–10; Grade III, scores of 11–20; and Grade IV, scores above 21. Cronbach’s α coefficient of this scale was 0.75.

#### Patient Health Questionnaire-9 (PHQ-9)

PHQ-9 includes nine items pertaining to DSM-IV criteria for major depressive disorder (MDD) [22]. Each item asks about depressive symptoms. It is rated on a 4-point scale (from 0 to 3) in the preceding two weeks. The overall score ranges from 0 to 27, with higher score indicating higher degree of depressive symptoms. The Korean version of the PHQ-9 has been validated in patients with migraine [17]. A cutoff score of 7 has been suggested as a score for differentiating MDD. Cronbach’s α coefficient of this scale was 0.894.

#### Generalized Anxiety Disorder-7 (GAD-7)

GAD-7 was designed to detect anxiety in primary care patients [23]. It consists of seven items pertaining to DSM-IV criteria for GAD. Each item asks about anxiety symptoms during the preceding two weeks. It is rated on a 4-point scale (from 0 to 3). The overall score ranges from 0 to 21, with a higher score meaning higher degree of anxiety symptoms. The Korean version of the GAD-7 has been validated in patients with migraine [18]. A cutoff score of 5 has been suggested as a score differentiating GAD. Cronbach’s α coefficient of this scale was 0.915.

#### Epworth Sleepiness Scale (ESS)

ESS is comprised of eight questions, each of them asking about the subject’s likelihood of dozing off or falling asleep in a particular situation that is common in daily life [24]. Thus, each ESS item score measures a particular “situational sleep propensity”. The sum of those item scores (i.e., total ESS score) measures the subject’s average sleep propensity across those different situations in daily life. Respondents used a four-point scale to score each of eight questions. A higher score indicates a higher subjective sleepiness. The Korean version of the ESS has been validated in patients with obstructive sleep apnea [19]. Cronbach’s α coefficient of ESS was 0.917.

#### Insomnia Severity Index (ISI)

Insomnia Severity Index (ISI) is a seven-item questionnaire that measures patient’s perception of insomnia severity [25]. Each of these ISI items is rated on a scale of 0–4. Its total score ranges from 0 to 28, with a higher score indicating a greater insomnia severity. The Korean version of the ISI has been validated in patients with sleep disorders [20]. A cutoff score of 15.5 has been suggested for discriminating patients with insomnia. Cronbach’s α coefficient of ISI was 0.92.

#### Migraine-Specific Quality of Life Questionnaire Version 2.1 (MSQ)

MSQ measures the impact of migraine on QOL over the previous four weeks across three dimensions: Role Function-Restrictive (RR), Role Function-Preventive (RP), and Emotional Function (EF) [26]. MSQ Version 2.1 consists of 14 questions, including 7 questions in the RR dimension, 3 questions in the PR dimension, and 4 questions in the EF dimension. MSQ has shown a high internal consistency, a moderate convergent validity, and a strong reliability [21]. Each MSQ question has six available answers (none, a little bit, some, a good bit, most of the time, and all the time) with scores of 1 to 6. Dimension scores are summed and rescaled to give a total score of 0 to 100. Higher score of MSQ indicates a better QOL. Cronbach’s α coefficients of this scale ranged from 0.86 to 0.96.

#### Statistical analysis

Statistical Package for Social Sciences (SPSS version 21.0) was used for data analysis. Descriptive statistics are presented as counts, percentages, means, and standard deviations. Independent *t* test, analysis of covariance, or Chi-square test was applied to compare two groups. To determine the relationship between various independent variables and PSS score, Pearson’s correlation coefficient was determined. Variables having significant correlation with PSS score were then included in multiple linear regression analyses with stepwise selection using entry and exit probabilities of 0.05 and 0.1, respectively. MSQ scores were not included as independent variables because they represented the overall outcome of events. Statistical significance was considered at *p* < 0.05.

## Results

### Baseline characteristics

Of 267 patients who visited our clinic, 40 were excluded from this study due to refusal to participate (*n* = 14), probable migraine (*n* = 10), serious medical or neurological disorders (*n* = 8), age older than 70 years (*n* = 5), age younger than 18 years (*n* = 2), and illiteracy (*n* = 1). Finally, 227 patients were eligible for this study. A total of 170 healthy controls were also included for this study. Demographic, socioeconomic, and clinical characteristics of participants are summarized in Table 1. Although migraine patients and controls had no significance difference in age, gender, employment status, household income, or body mass index, education level in migraine patients was significantly (*p* < 0.001) lower than that in controls. A total of 208 (91.6%) migraine patients had migraine without aura while 103 (45.4%) patients had CM.

**Table 1:** Demographic, socioeconomic, and clinical characteristics of the participants

Characteristic	Mean ± SD (range) or number (%)		*P* value*
	Patients with migraine (*n* = 227)	Controls (*n* = 170)	
Age, years	42.4 ± 12.5 (18–68)	40.8 ± 11.5 (19–70)	0.182
Gender, female	190 (83.7)	135 (79.4)	0.294
Education, years	12.7 ± 3.1 (6–20)	15.0 ± 2.4 (6–20)	<0.001
Employment, yes	134 (59.0)	109 (64.1)	0.349
Household income, at least 3 million KRW/month	165 (72.7)	130 (76.5)	0.418
Body mass index	22.7 ± 3.2 (16.9–35.9)	22.9 ± 3.7 (15.2–40.1)	0.422
Concurrent medical disease, yes	102 (44.9)		
Type of migraine			
Migraine with aura	19 (8.4)		
Migraine without aura	208 (91.6)		
Migraine chronicity, CM	103 (45.4)		
Age at onset, years	31.1 ± 12.3 (10–60)		
Duration of migraine, years	11.3 ± 9.2 (0.3–42)		
VAS	1.8 ± 2.4 (0–10)		
Medication overuse headache	27 (11.9)		
Family history of migraine	139 (61.2)		
Associated symptoms			
Nausea/vomiting	193 (85)		
Photophobia	104 (45.8)		
Phonophobia	141 (62.1)		
Osmophobia	103 (45.4)		
Allodynia	40 (17.6)		
MIDAS	24.7 ± 28.9 (0–181)		
ESS	6.0 ± 4.2 (0–18)		
ISI	9.0 ± 6.2 (0–26)		
MSQ			
Role Function-Restrictive	58.0 ± 24.0 (0–100)		
Role Function-Preventive	69.8 ± 24.0 (0–100)		
Emotional Function	73.5 ± 25.4 (0–100)		

*KRW* Korean Won, *CM* chronic migraine, *VAS* Visual Analog Scale, *MIDAS* Migraine Disability Assessment Scale, *MSQ* Migraine-Specific Quality of Life Questionnaire Version 2.1, *ESS* Epworth Sleepiness Scale, *ISI* Insomnia Severity Index

*Independent t test or Chi-square test was applied

### Levels of perceived stress

Levels of perceived stress in migraine patients compared to those in controls are summarized in Table 2. While the mean PSS score adjusted for education was significantly (*p* < 0.001) higher in migraine patients than that in controls, the score adjusted for education, depression, and anxiety in migraine patients was not significantly different from that in controls. Regarding migraine chronicity, only CM patients had higher (*p* < 0.05) mean PSS scores than controls after controlling for education, depression, and anxiety.

**Table 2:** Perceived stress, depression, and anxiety in migraine patients compared with controls

	Mean ± SD (range)			
	Migraine patients	Episodic migraine	Chronic migraine	Healthy controls
	(*n* = 227)	(*n* = 124)	(*n* = 103)	(*n* = 170)
PSS	18.6 ± 6.3 (6–36)^c^	17.1 ± 6.2 (6–34)	20.5 ± 5.8 (7–36)^c,^*	15.9 ± 5.5 (0–29)
PHQ-9	6.8 ± 5.5 (0–27)^c^	5.6 ± 5.0 (0–20)^b^	8.3 ± 5.7 (0–27)^c^	3.7 ± 4.3 (0–18)
GAD-7	5.3 ± 5.1 (0–21)^c^	4.4 ± 4.8 (0–19)^a^	6.3 ± 5.4 (0–21)^c^	3.3 ± 3.5 (0–19)

Analysis of Covariance controlling for education between groups with migraine and controls. ^a^ *p* < 0.05, ^b^ *p* < 0.01, ^c^ *p* < 0.001

Analysis of Covariance controlling for education, depression, and anxiety between groups with migraine and controls. *p < 0.05

PSS: Perceived Stress Scale, PHQ-9: Patient Health Questionnaire-9, GAD-7: Generalized Anxiety Disorder-7

### Factors associated with PSS score

Variables associated with PSS score in migraine patients by univariate analyses are listed in Table 3. CM, earlier onset of migraine, higher intensity of headache, phonophobia, allodynia, higher scores of MIDAS, PHQ-9, GAD-7, ESS, and ISI were associated with PSS score. However, gender, education, or socioeconomic factor was not associated with perceived stress.

**Table 3:** Variables associated with the PSS score by univariate analyses in migraine patients

Variable	*P* value (*r*)^a^
CM	<0.001 (−0.271)
Onset	0.047 (−0.132)
VAS	<0.001 (0.313)
Phonophonia	0.004 (−0.192)
Allodynia	<0.001 (−0.238)
MIDAS	<0.001 (0.405)
PHQ-9	<0.001 (0.683)
GAD-7	<0.001 (0.670)
ESS	0.003 (0.195)
ISI	<0.001 (0.488)

*PSS* Perceived Stress Scale, *CM* chronic migraine, *VAS* Visual Analog Scale, *MIDAS* Migraine Disability Assessment Scale, *PHQ-9* Patient Health Questionnaire-9, *GAD-7* Generalized Anxiety Disorder-7, *ESS* Epworth Sleepiness Scale, *ISI* Insomnia Severity Index

^a^Pearson’s correlation was applied

### Predictors for PSS score

Predictors for PSS score in migraine patients based on multivariate analyses are summarized in Table 4. The strongest predictor was the GAD-7 score (*β* = 0.358, *p* < 0.001), followed by the PHQ-9 score (*β* = 0.304, *p* < 0.001), the ISI score (*β* = 0.154, *p* = 0.005), and CM (*β* = −0.104, *p* = 0.027). These four variables explained 54.2% of the variance in PSS scores. Variance inflation factor (VIF) was less than 10 for all variables, suggesting that they exerted independent effects without redundancy.

**Table 4:** Predictors for the PSS score in migraine patients by multivariate analyses

Variable	Standardized coefficients *(β)*	*P* value	Collinearity (VIF)	Adjusted *R* ^*2*^
Constant		<0.001		0.542
GAD-7	0.358	<0.001	2.307	
PHQ-9	0.304	<0.001	2.765	
ISI	0.154	0.005	1.433	
CM	−0.104	0.027	1.066	

*PSS* Perceived Stress Scale, *GAD-7* Generalized Anxiety Disorder-7, *PHQ-9* Patient Health Questionnaire-9, *ISI* Insomnia Severity Index, *CM* chronic migraine

### Relationship between PSS score and quality of life

**Fig. 1:**
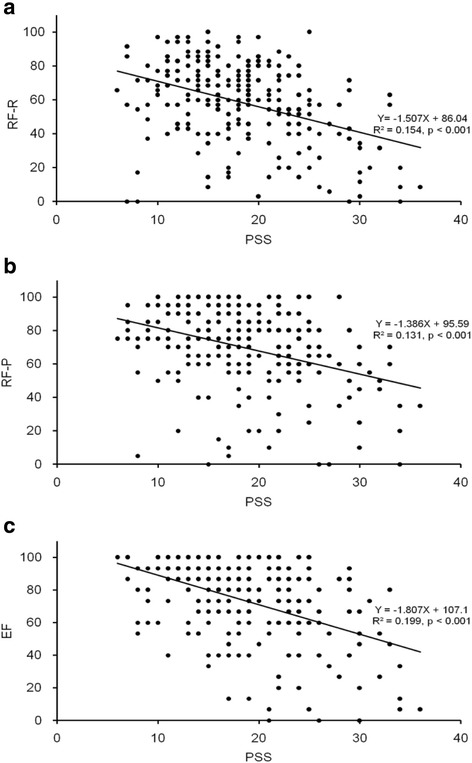
Relationship between the PSS score and the MSQ score. Three dimensional scores of the MSQ (**a**, **b**, and **c**) were inversely correlated with the PSS score (*p* < 0.001). PSS: Perceived Stress Scale, MSQ: Migraine-Specific Quality of Life Questionnaire Version 2.1, RF-R: Role Function-Restrictive, RF-P: Role Function-Preventive, EF: Emotional Function

The relationship between PSS score and three-dimensional scores of MSQ is shown in Fig. 1. RR, RP, and EF dimension scores of MSQ were inversely correlated with PSS score (*p* < 0.001).

## Discussion

Our study revealed that the level of perceived stress was significantly higher in CM patients than that in controls. Although several factors including clinical and psychosomatic factors were associated with perceived stress, our data demonstrated that CM appeared to be a critical factor for perceived stress. Perceived stress was correlated well with migraine-specific QOL.

While a higher level of perceived stress has been previously reported in migraine patients compared to that in healthy controls in two studies [6, 7], there was no difference in mean PSS score between migraine patients and controls after controlling for depression and anxiety in this study. This reveals that depression and anxiety are major determinants of perceived stress in migraine patients and controls. It is known that stressful events can cause depression and anxiety. In response to stress, corticotropin releasing factor (CRF) regulates the activity of hypothalamic-pituitary-adrenal (HPA) axis and triggers changes in serotonin receptors [27]. CRF is also known to influence anxiety responses with CRF receptor 1 being particularly important [28]. In a cohort study, it has been found that stressful events contribute to comorbidity of migraine and major depression [29]. Depression and anxiety can also aggravate stressful conditions. In epilepsy patients, depression and anxiety have direct effect on perceived stress [30]. Under these circumstances, depression and anxiety are not likely to be unique for perceived stress in patients with migraine.

After investigating the relationship between migraine chronicity and perceived stress, it was found that CM patients had higher levels of perceived stress than controls. CM was selected as a critical factor for perceived stress after adjusting for depression, anxiety, and insomnia by multivariate analyses. It has been reported that CM patients are more likely to have depression, anxiety, sleep problems, and poor QOL compared to EM patients [31, 32]. These conditions might induce stressful conditions in CM patients to some extent. Our data demonstrated that CM appeared to be a migraine-specific factor for perceived stress. Stressful life events are likely to trigger migraine events [8, 9]. They might be risk factors for CM [11]. Repeated stress may lead to functional and structural alteration in the brain network. These changes in brain states may occur as a result of repeated migraine attacks through maladaptive coping mechanisms [33]. The cascade of these effects can lead to further deterioration of adaptation, causing transformation or chronification of the disease [33]. Therefore, clinicians should identify perceived stress by counseling migraine patients. They need to modify perceived stress through pharmacological or non-pharmacological interventions such as cognitive behavioral therapy and biofeedback to avoid transformation or chronification of migraine [34].

Our results revealed that the level of perceived stress was significantly associated with the role function and emotional function of migraine patients. Chronic stress may trigger migraine attacks [8, 9] or induce CM [11, 33], subsequently restricting or preventing participation in social or work related activities [35]. Therefore, chronic stress might affect emotions of migraine patients. That is the reason why clinicians should identify and modify stress.

Our study has some limitations. First, subjects were from a single tertiary hospital. Therefore, our results cannot be generalized. Second, this was a cross-sectional study. Causal relationships between variables could not be confirmed. A longitudinal study is recommended to verify the causal relationship between perceived stress and CM. Third, the level of perceived stress was measured for the preceding month. Therefore, state of stress over a month was unknown. A long-term observational study is needed to evaluate the impact of CM on perceived stress.

## Conclusions

In conclusion, it was found that the level of perceived stress was significantly higher in CM patients than that in controls. Among several factors associated with perceived stress, CM appeared to be a critical factor for perceived stress. Significant negative correlations between perceived stress and migraine-specific QOL was found in this study.

## Abbreviations

ASC: Allodynia Symptom Checklist
CM: Chronic migraine
CRF: Corticotropin releasing factor
EF: Emotional Function
EM: Episodic migraine
ESS: Epworth sleepiness scale
GAD: Generalized Anxiety Disorder
HPA: Hypothalamic-pituitary-adrenal
ISI: Insomnia severity index
MDD: Major depressive disorder
MIDAS: Migraine Disability Assessment Scale
MSQ: Migraine-Specific Quality of Life Questionnaire Version 2.1
PHQ: Patient Health Questionnaire
PSS: Perceived stress scale
QOL: Quality of life
RP: Role Function-Preventive
RR: Role Function-Restrictive
VAS: Visual analog scale
VIF: Variance inflation factor
